# Research on the coupling coordination characteristics and convergence of digital finance and regional sustainable development: evidence from Chinese city clusters

**DOI:** 10.1038/s41598-024-66890-5

**Published:** 2024-07-11

**Authors:** Qiguang An, Yongkai Wang, Qinggang Meng, Ruoyu Wang, Qian Xie

**Affiliations:** 1 School of Statistics and Mathematics, Shandong University of Finance and Economics, Jinan, Shandong China; 2 Department of Reproductive Medicine, Central Hospital Affiliated to Shandong First Medical University, Jinan, Shandong China

**Keywords:** Digital finance, Coupling coordination, Regional sustainable development, Kernel density, Spatial convergence, Environmental social sciences, Engineering, Mathematics and computing

## Abstract

The study examines the digital finance (DF) and regional sustainable development (RSD) across 90 cities within six major city clusters in China over the period from 2011 to 2020. By constructing an evaluation index system for DF and RSD, we employed the entropy value method to assess their levels, and the coupling coordination degree (CCD) model to evaluate their interplay. Our analysis extended to temporal and spatial disparities, distribution dynamics, and the convergence of CCD through kernel density estimation, Markov chain analysis, $\sigma$-convergence, and $\beta$-convergence techniques. The results indicate a consistent upward trend in CCD, yet it remains at a low level with pronounced regional disparities and temporal characteristics. The kernel density distribution’s central tendency has shifted rightward progressively, albeit with a decelerating pace annually. The Markov transition probability matrix suggests a stable CCD across various levels, hinting at “club convergence”. Furthermore, both $\sigma$-convergence and $\beta$-convergence analyses reveal significant convergence trends in CCD, enhanced by economic growth factors. Using the Quadratic Assignment Procedure (QAP) method, we found that regional economic growth disparities significantly influence the CCD’s regional variances.

## Introduction

In the era of rapid global development, sustainable development has emerged as a critical strategy for achieving long-term prosperity and stability. This is especially pertinent for China, where sustainable development is central to national growth objectives^1,2^. The 20th National Congress of the Communist Party of China underscored the necessity of “advancing human-centric new urbanization,” highlighting it as a crucial pathway toward achieving sustainable development goals. Central to this strategy is the development of city clusters, which aim to drive synchronized progress among large, medium, and small cities, as well as small towns. The “14th Five-Year Plan” for new urbanization in China places significant emphasis on the structured promotion of city cluster development. This plan aims to enhance the population and economic capacities of these clusters, positioning them as powerhouses and catalysts for regional sustainable development (RSD). Consequently, city clusters are integral to China’s urbanization and regional development strategies, acting as pivotal forces in fostering overall RSD.

City clusters, defined as geographically proximate urban areas interconnected through economic, social, and infrastructural linkages, serve as engines of economic growth and innovation. The development of city clusters is a strategic move to promote balanced regional development, reduce urban–rural disparities, and achieve high-quality economic growth. In China’s pursuit of new urbanization, the coordinated development of city clusters is expected to play a central role in achieving sustainable development objectives, including economic efficiency, social equity, and environmental sustainability.

Finance, recognized as the lifeblood of economic development, plays an irreplaceable role in promoting high-quality economic and societal progress. In the contemporary era characterized by big data, cloud computing, artificial intelligence, and other advanced technologies, digital finance (DF) has undergone significant transformation, revolutionizing the financial landscape. DF encompasses a wide array of financial services delivered through digital platforms, including mobile payments, online banking, digital lending, crowdfunding, and blockchain-based financial services. These advancements have made financial services more accessible, efficient, and inclusive, particularly for underserved populations and regions. By leveraging technology, DF reduces transaction costs, enhances financial literacy, and broadens financial inclusion, contributing to sustainable economic growth and development. DF also has the potential to enhance inclusive finance by reducing customer acquisition costs and providing diverse risk management and control mechanisms. It supports financial inclusion by offering services to those who previously had limited or no access to traditional financial systems. Additionally, DF contributes to environmental sustainability by lowering carbon emissions, thereby improving environmental quality. For instance, digital transactions reduce the need for paper-based processes and physical transportation, cutting down on resource consumption and emissions. Thus, DF is a critical enabler of RSD, offering pathways to integrate financial inclusivity and environmental sustainability. This multifaceted impact underscores the significance of DF in fostering high-quality economic and societal progress, making it an essential component of modern economic systems.

Despite its transformative potential, the relationship between DF and RSD remains underexplored, particularly within the context of city clusters. The interplay between DF and RSD is complex and multifaceted, involving various economic, social, and environmental dimensions. Understanding this relationship is crucial for designing policies that leverage DF to promote sustainable development at the regional and national levels. This study addresses this gap by examining the coupling coordination degree (CCD) between DF and RSD across six major city clusters in China from 2011 to 2020. By constructing an evaluation index system for both DF and RSD, this research employs the entropy value method to assess their respective levels and the CCD model to evaluate their interplay. The study delves into the temporal and spatial disparities, distribution dynamics, and convergence of CCD using kernel density estimation, Markov chain analysis, $\sigma$-convergence, and $\beta$-convergence techniques.

The primary objectives of this research are to: assess the CCD between DF and RSD within Chinese city clusters; analyze the spatial and temporal evolution of CCD; investigate the convergence trends of CCD; and understand the dynamic evolution of CCD in the context of new urbanization. The significance of this study lies in its potential to fill several critical gaps in the existing literature. First, it provides a comprehensive assessment of the CCD between DF and RSD at the city cluster level, offering a nuanced understanding of their interplay across different regions and time periods. Second, it employs a robust methodological framework, incorporating the entropy value method, CCD model, kernel density estimation, Markov chain analysis, $\sigma$-convergence, and $\beta$-convergence techniques to analyze the dynamic evolution and convergence trends of CCD. Third, it explores the role of DF in promoting RSD, highlighting its potential to enhance financial inclusion, economic efficiency, and environmental sustainability.

The study’s findings will offer a theoretical foundation and policy recommendations for advancing the CCD of DF and RSD, providing insights into enhancing RSD through DF. By addressing the significant regional disparities and temporal characteristics in CCD, the research aims to inform strategies that promote balanced and inclusive growth across city clusters. This study not only contributes to the theoretical understanding of DF and RSD interplay but also provides practical implications for policymakers aiming to harness DF as a tool for sustainable urban development.

## Literature review

The relationship between DF and RSD has attracted considerable scholarly attention, spanning several key domains. This review synthesizes current findings, categorizing them into four main areas: environmental sustainability, corporate sector advancement, agricultural development, and broader dynamics between DF and RSD.

1. DF and environmental sustainability. One significant area of research focuses on DF’s influence on environmental betterment, particularly in carbon emission mitigation. Wu et al.^3^revealed a U-shaped correlation between DF and carbon efficiency in Chinese city clusters, indicating that initial increases in DF may lead to higher emissions, but further advancements enhance carbon efficiency. Similarly, Wang and Guo^4^ use data from 272 Chinese cities and a spatial econometric model, supporting DF’s role in reducing urban carbon emissions. Zhou et al.^5^ found that DF, especially its depth of use, can reduce industrial exhaust emissions and exhibit significant green emission effects. Yuan et al.^6^ analyzed the impact of DF on ecological environment quality based on county-level panel data in China, concluding that industrial agglomeration and structural transformation are key mechanisms by which DF hinders ecological environment performance. Li et al.^7^ explored the spatial relationships among DF, financing constraints, and green technological innovation using the Spatial Durbin Model, finding that DF positively impacts green technological innovation by reducing financial constraints and enhancing information exchange between traditional businesses and banks.
2. DF and corporate sector advancement. Research in the corporate sector investigates DF’s contribution to the high-quality development (HQD) of enterprises. Xie et al. ^8^ analyzed the connection between DF and enterprise HQD, particularly under financing constraints, focusing on state-owned firms listed on China’s stock exchanges. They found that DF enhances HQD by easing financing restrictions. Similarly, Xie and Liu^9^ assessed DF’s impact on the HQD of small and medium-sized enterprises (SMEs), concluding that DF is vital for improving SMEs’ development quality through efficient financial resource allocation. Huang et al.^10^, using data from Chinese A-share listed companies, explored the impact of regional DF development on corporate investment efficiency. The analysis revealed that DF significantly improves corporate investment efficiency, with reducing financing constraints and stimulating corporate innovation being the main mechanisms. Tang and Geng^11^, using data from Chinese A-share listed companies, examined the impact of DF development on corporate debt financing costs. They found that the development of DF significantly reduces corporate debt financing costs, with the breadth of coverage having the most pronounced effect, and noted heterogeneity across different regions and industries.
3. DF and agricultural development. In the agricultural sector, research has examined DF’s role in fostering high-quality growth. Zhang et al.^12^ demonstrated that DF promotes superior agricultural development through both direct and indirect means, influenced by factors such as mechanization levels. Their study, using structural equation modeling and provincial data from China, revealed the positive impact of DF on agricultural sustainability. Similarly, Sun and Zhu^13^ constructed an index to empirically examine DF’s impact on rural sustainable growth in China, highlighting significant contributions through economic efficiency, urban–rural dynamics, and ecological sustainability. These studies emphasize the importance of DF in bridging the urban–rural divide and promoting sustainable agricultural practices.
4. Broader dynamics between DF and RSD. Beyond specific sectors, broader studies have investigated the synergistic dynamics between DF and RSD. Li et al.^14^ used data from 30 provinces in China to analyze the coupling and coordinated development between green finance and the SDGs system through the CCD model, finding an M-shaped upward trend in most regions. Similarly, Yang et al.^15^conducted an empirical analysis of the coupling and coordinated development of new urbanization and ecological welfare performance within China’s Chengdu-Chongqing Economic Circle using the CCD model and the spatio-temporal geographically weighted regression model. Cui et al.^16^ used the Multiscale Geographically Weighted Regression model to study the spatiotemporal relationship between industrial electricity consumption and economic growth at the prefecture-level city level in China. Shen et al.^17^ conducted an empirical study on the spatial coupling relationship and interaction mechanism between green urbanization and tourism competitiveness in 734 counties in the Yellow River Basin in China.

Despite these advancements, there remains a notable gap in research focusing on the characteristics and convergence of the DF and RSD coupling at the micro or urban scale. The existing literature primarily addresses broader regional or sectoral analyses, leaving the detailed urban dynamics less explored. This gap suggests a potential area for further investigation, particularly in understanding how DF can be effectively leveraged to support sustainable urban development at a granular level. Future research should aim to fill this gap by examining the CCD between DF and RSD within city clusters, analyzing spatial and temporal disparities, distribution dynamics, and convergence trends. Such studies will provide valuable insights into enhancing RSD through DF and inform policy decisions to promote balanced and inclusive growth across city clusters.

**Figure 1:**
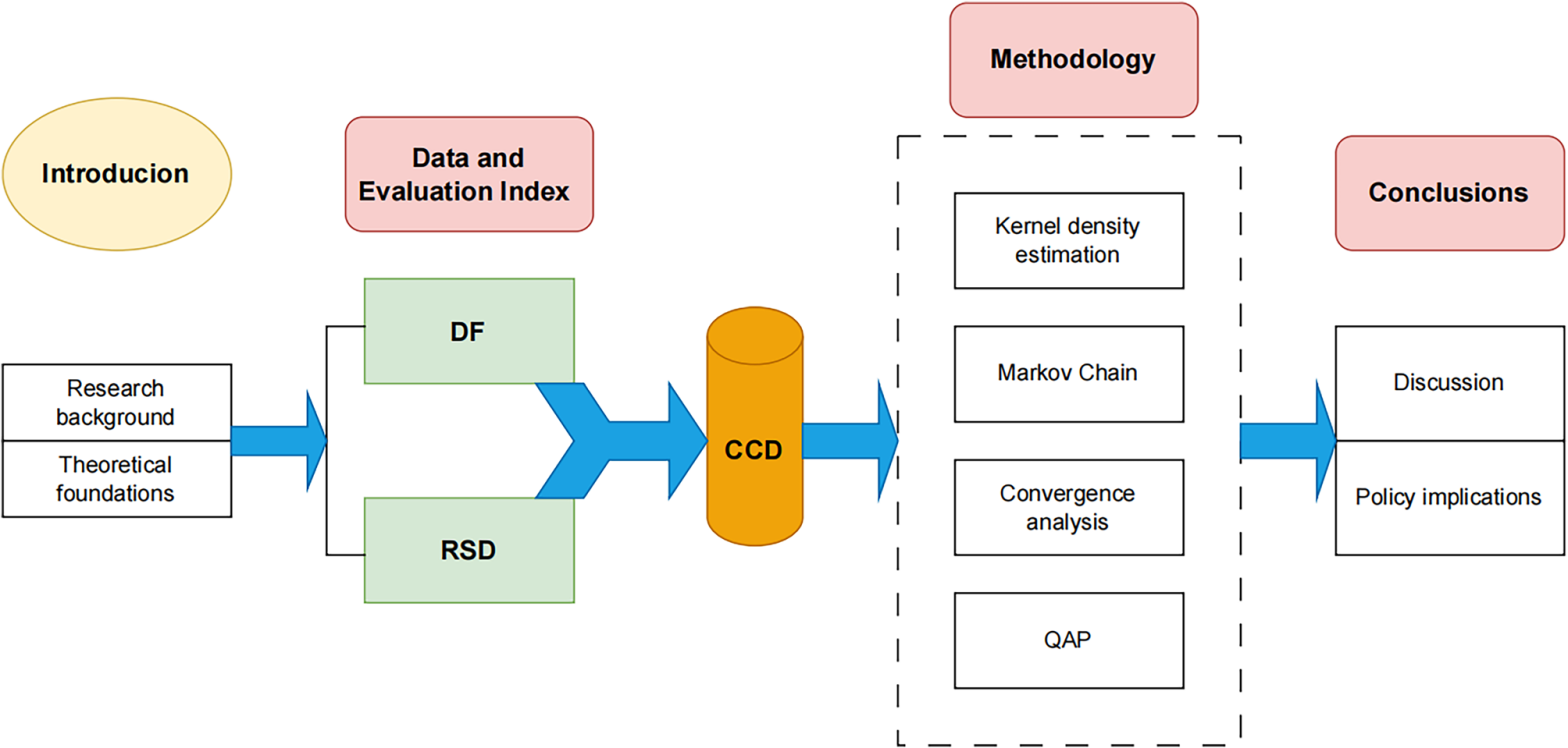
The framework of this paper.

With spatial and geographical proximity, frequent interaction of economic and social activities, and interpenetration of infrastructures, city clusters are the most dynamic and growth-potential geographic regions in China’s economic development. Because of this, this paper will study the coupling and coordination characteristics and convergence of the two systems based on city clusters. Initially, separate evaluation index systems for DF and RSD will be established, with the entropy value method deployed to gauge the developmental levels of both systems. Subsequently, the CCD model will be utilized to assess the interaction between the two systems. Further, an integrated method comprising kernel density estimation, Markov chain analysis, $\sigma$-convergence, and $\beta$-convergence will be employed to analyze the temporal and spatial disparities, the evolving distribution dynamics, and the convergence patterns of the CCD. Lastly, the Quadratic Assignment Procedure (QAP) method will be applied to empirically investigate the determinants of regional variability in coupling coordination, thus providing a comprehensive understanding of the intricate interplay between DF and RSD. The framework of this paper is shown in Fig. 1.

## The evaluation index system

### The theoretical interaction mechanism of DF and RSD

DF emerges from the integration of cutting-edge technologies like cloud computing and big data, representing a novel business paradigm. In this model, traditional financial entities and internet firms utilize advancements in the internet, big data, blockchain, and artificial intelligence to drive innovation in financial products. This convergence facilitates a more efficient, accessible, and flexible financial services landscape, enabling a transformative approach to meeting diverse financial needs and enhancing economic inclusivity. The genesis of DF is often linked to the inception of Alipay, in 2004, and since 2012, P2P lending, third-party payments, Internet credit, and other Internet financial businesses have proliferated in China, and academics and the financial sector usually regard the launch of Balance Bao in 2013 as the first year of China’s digital financial development. In February 2023, Chinese officials released the “Overall Layout Plan for the Construction of Digital China”. This strategic document explicitly advocates for a comprehensive digital transformation of the financial sector. It envisions a future where the digital economy not only enhances financial services but also offers improved service quality and greater scope for financial innovation. This transformation is aimed at supporting the development of the real economy, suggesting a strategic direction where digital integration plays a key role in fostering economic growth and financial sector innovation.

DF and RSD are interconnected through a dialectical relationship that fosters mutual enhancement, coordination, and symbiosis. DF, powered by the latest technological advancements, plays a pivotal role in driving RSD. It achieves this through its broad coverage and deep utilization, characteristics inherent to its tech-driven and inclusive essence. DF extends the reach of financial services, channeling financial resources efficiently to vital yet under-served sectors of the economy and society, such as small and micro businesses, agriculture, and rural areas. This expansion of financial services not only meets diverse economic needs but also fuels sustainable regional development by enhancing financial accessibility. Conversely, the progress of RSD serves as a crucial bedrock for the evolution of DF. As DF emerges from cutting-edge information technologies, its growth is contingent upon the ongoing evolution and enhancement of economic and societal frameworks. The advancement of new-generation information technologies and infrastructures, like the internet, provides the necessary groundwork for DF’s inception and expansion. Moreover, the spread, evolution, and adoption of DF are closely tied to the sustainable development of the economy and society, highlighting a reciprocal relationship where each domain fosters and reinforces the other’s growth.

Based on this, we put forward the following hypotheses: (1) The development of DF has a positive supporting effect on RSD. (2) RSD plays a positive role in promoting DF.

### Evaluation index system construction

In the academic realm, a standardized evaluation index system for DF and RSD has yet to be established, leading to varied indicators being developed by researchers based on their specific objectives. For DF, a notable example is the “Digital Inclusive Finance Index” crafted by the Digital Finance Research Center at Peking University^18,19^, which encapsulates DF’s evolution across three facets: usage depth, coverage breadth, and digitization level, making it a benchmark in the field. This study adopts these three indicators to delineate DF’s landscape in the six major city clusters in China. Utilizing the entropy value method, it quantifies DF’s level, ensuring an analytical framework that not only captures the essence of digital financial development but also aligns with the nuanced complexities of regional sustainable growth.

Scholarly work on formulating an evaluation index system for RSD is extensive and multifaceted. This body of research can be categorized into several key strands. Initially, a prominent approach aligns with the new development paradigm, as noted by Zheng and He^20^ and Li et al.^21^, which frames evaluation systems around the “Five Development Concepts”—innovation, coordination, green development, openness, and sharing. Extending this framework, Deng et al.^22^incorporated an “economic” dimension, thus expanding the framework to six dimensions to assess RSD in the Jing-Jin-Ji metropolitan area. Similarly, Li and Liu^23^ augmented this model with a “safety” dimension, creating a six-faceted evaluation system for China’s marine fisheries’ sustainable development, embracing openness, innovation, coordination, greenness, sharing, and safety. From the perspective of economic growth, another research vein employs metrics like total factor productivity^24^, green total factor productivity^25,26^, and labor productivity^27^ to gauge RSD. Although these studies benefit from straightforward indicator computations, they often encounter challenges such as limited representativeness, one-dimensionality, and inability to fully capture the essence of RSD. A third perspective emphasizes multidimensional approaches, particularly green development. For instance, Zhang et al.^28^ applied the undesirable-SE-SBM model to assess green development efficiency. Drawing on the work of Yin et al.^29^, this paper proposes an RSD evaluation framework comprising five primary dimensions: innovation capacity, economic vitality, shared development, open development, and green development. This framework includes 18 specific indicators, such as the industrial smoke and dust emissions, aiming to provide a comprehensive depiction of RSD. The detailed assessment framework for DF and RSD is presented in Table 1.

**Table 1:** Assessment framework of DF and RSD.

System	Target layer	Specific Indicators	Unit	Direction	Weight
DF	Usage depth	Usage depth	score	+	0.3307
	Coverage breadth	Coverage breadth	score	+	0.3305
	Digitization level	Digitization level	score	+	0.3389
RSD	Innovation power	Investment in science and technology	%	+	0.0883
		IRIEC Trademark Registration Number Score	score	+	0.0266
		human capital	%	+	0.1500
		IRIEC Patent License Number Score	score	+	0.0177
	Economic vitality	Industrial progression level	%	+	0.0709
		Regional GDP per capita	yuan	+	0.0572
		Industrial Optimization Level	score	-	0.0140
	Shared development	Parkland area per capita	square meters	+	0.0449
		Rural income disparity level	%	+	0.0228
		Public library book volume	volumes/person	+	0.1069
		urbanization rate	%	+	0.0656
		Healthcare accessibility index	number of hospital beds per 10,000	+	0.0583
	Open development	IRIEC City FDI Attraction Score	score	+	0.0236
		Foreign enterprise count	number of enterprises	+	0.1580
		urban attraction	%	+	0.0798
	Green development	Sulfur dioxide emission level	tons/GDP	-	0.0074
		Non-hazardous treatment rate of domestic waste	%	+	0.0073
		Industrial smoke and dust emissions	tons/GDP	-	0.0007

### Data sources

**Figure 2:**
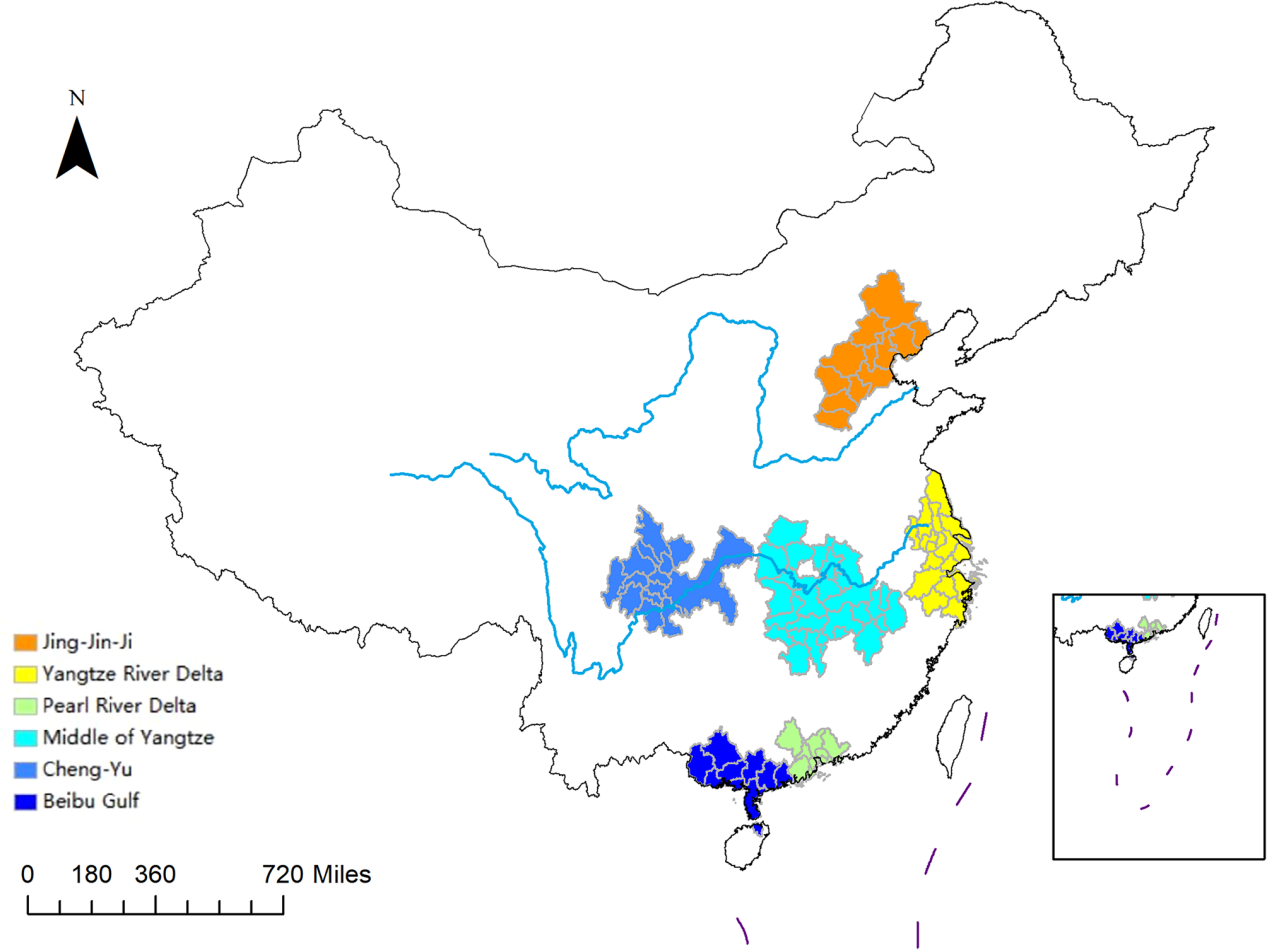
The distribution of the six urban clusters in China. The map is based on the standard map with review number GS (2019) 1822 downloaded from the Standard Map Service website of the Ministry of Natural Resources (http://bzdt.ch.mnr.gov.cn/), with no modifications to the base map.

Building on the research by Feng et al.^30^ and Wang and Li^31^, as well as the strategic demarcations and roles outlined in the “14th Five-Year Plan” for new urbanization, this study selects the following city clusters for analysis: Jing-Jin-Ji, Yangtze River Delta, Guangdong-Hong Kong-Macao Greater Bay Area (represented as Pearl River Delta due to the exclusion of Hong Kong SAR and Macao SAR for data limitations), the Middle Yangtze, Chengdu-Chongqing (Cheng-Yu), and Beibu Gulf city clusters. This selection encompasses 90 cities across these six national-level clusters. Figure 2 illustrates the geographical distribution of these six major city clusters.

The Digital Inclusive Finance Index, inaugurated in 2011, serves as a critical data source for this study. Given the unavailability of certain city-level data for 2021 to 2023, the timeframe of 2011–2020 has been selected for analysis. The metrics for digital finance are directly sourced from the aforementioned index, while the indicators for RSD—such as invention patents, trademark registrations, and foreign investment appeal—are obtained from Peking University’s Research Center for Enterprise Big Data (IRIEC). Additional data for the study are extracted from the CSMAR regional economic database, the China Urban Statistical Yearbook, and the CNRDS database. For the years and cities where data are incomplete, efforts were made to manually compile the necessary information from regional statistical offices and government reports. In cases of isolated data gaps, interpolation methods have been employed to estimate the missing values, ensuring a comprehensive and consistent dataset for the analysis.

## Methodology

### Entropy value method

The entropy value method is highly regarded for its objectivity and scientific rigor in dynamic comprehensive evaluations. Its core principle involves utilizing the information entropy of each index to determine its weight, and then calculating the comprehensive evaluation result via a weighted average. This method involves several key steps:

To address the issue of inconsistent measurement units among indicators, which could skew comparisons, the evaluation index data for DF and RSD must first undergo standardization. This process adjusts the scale of the indicators to a uniform metric, mitigating potential scale-related discrepancies. The standardization formulas for positive and negative indicators are as follows:

1 $$ {\text{Positive indicators}}:r_{ij} = \frac{{a_{ij} - \min (a_{ij} )}}{{\max (a_{ij} ) - \min (a_{ij} )}} $$

2 $$ {\text{Negative indicators}}:r_{ij} = \frac{{\max (a_{ij} ) - a_{ij} }}{{\max (a_{ij} ) - \min (a_{ij} )}} $$

where $a_{ij}$ represents the original value of the indicator, while $r_{ij}$ signifies the standardized, dimensionless value. The terms max($a_{ij}$) and min($a_{ij}$) denote the maximum and minimum values of the indicator $j$ across all entities being evaluated, respectively. Following standardization, the entropy weight method is employed to ascertain the weight of each indicator, enhancing the accuracy of the comprehensive evaluation. The comprehensive development index for each system is then computed using the formula:

3 $$ U_{i} = \sum\limits_{j = 1}^{n} {w_{j} r_{ij} } $$

where $U_{i}$ denotes the comprehensive development level of the system, $w_{j}$ represents the weight of indicator $j$, and $n$ is the total number of indicators within the system. This approach ensures that the comprehensive index reflects the relative importance of each indicator, providing a nuanced and balanced assessment of the development level of each system.

### Dual-system coupling coordination model

In physics, the concept of coupling describes how two or more systems interact and influence each other. The CCD model quantifies the level of coordination between different systems. It has been widely applied across various disciplines, including economic, social, and environmental development. In economics, this model is primarily used to analyze the coordinated development levels among multiple systems, such as the interaction between the ecological environment, economy, and human society. For instance, it measures the interplay between ecology and economy^32,33^. Furthermore, it has been extensively utilized in research on green finance^34,35^ and sustainable development^36^.

This paper introduces the CCD model to assess the coupling and coordination between DF ($U_{1}$) and RSD ($U_{2}$).The model aims to evaluate the coordination effects and the dynamic evolution of the relationship between DF ($U_{1}$) and RSD ($U_{2}$). The mathematical formulation of the model is as follows:

4 $$ C = \sqrt {\frac{{U_{1} \times U_{2} }}{{\left( {\frac{{U_{1} + U_{2} }}{2}} \right)^{2} }}} $$

5 $$ T = \frac{{U_{1} \times U_{2} }}{2} $$

6 $$ D = \sqrt {C \times T} $$

In these equations, $U_{1}$ and $U_{2}$ represent the respective levels of DF and RSD. $C$ signifies the system’s harmonization level, with values ranging from 0 to 1, reflecting the extent of synergy between DF and RSD. $T$ is the combined evaluation score of the two systems.$D$ quantifies the overall coupling effect, also within a range of 0 to 1. A higher $D$ value indicates a stronger coupling and a more synergistic relationship, promoting mutual and coordinated development between DF and RSD.

**Figure 3:**
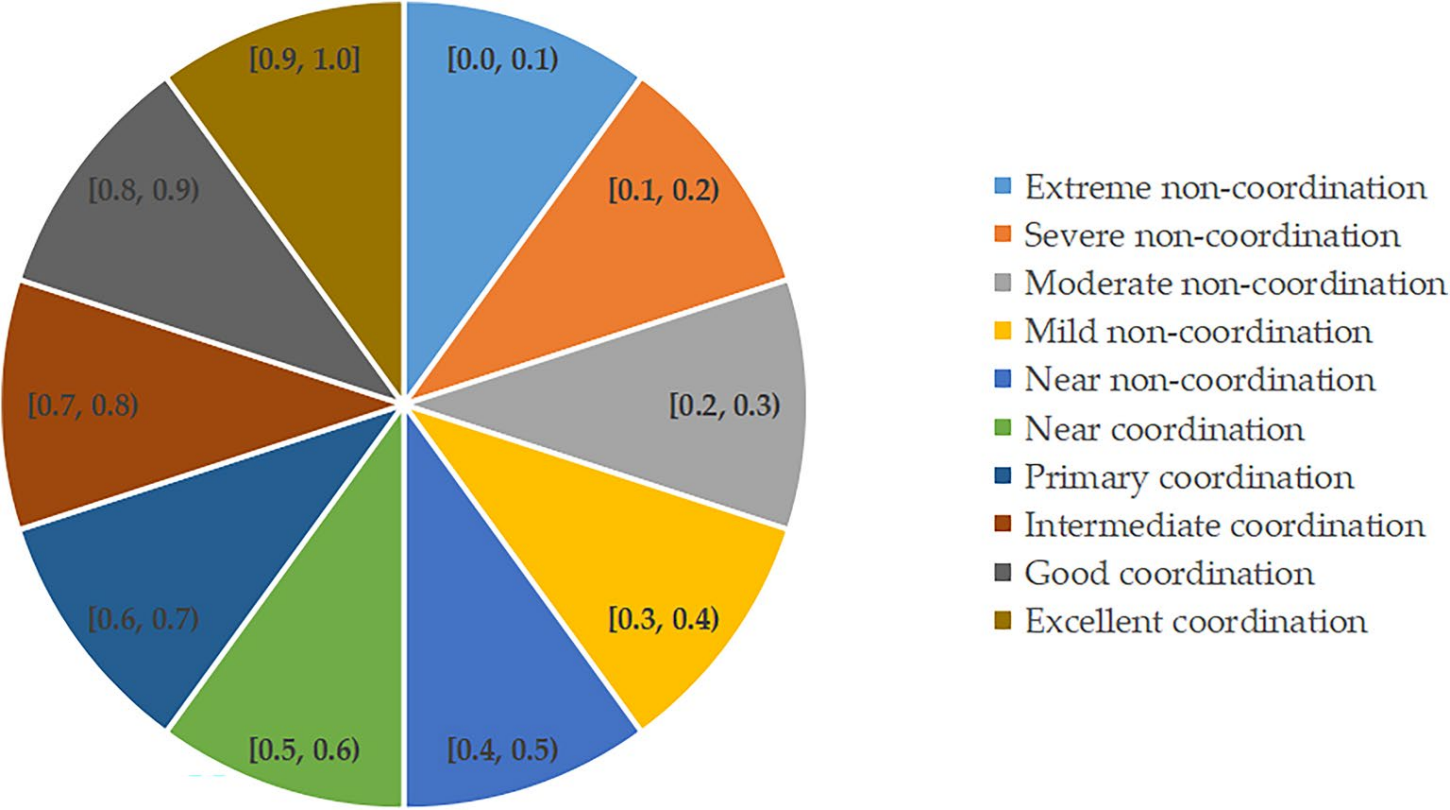
The Classification of CCD.

To depict the CCD between DF and RSD more vividly, this study adopts a classification scheme inspired by Hou et al.^37^, dividing the CCD into 10 distinct grades. These grades provide a nuanced view of the interaction dynamics, facilitating a deeper understanding of how these systems co-evolve and influence each other. The classification of these grades will be illustrated in Fig. 3, offering a detailed visual representation.

### Kernel density estimation

This study utilizes the kernel density estimation method to examine the distributional dynamics and variation in the CCD between DF and RSD across six major city clusters in China. The kernel density estimation, a non-parametric way to estimate the probability density function of a random variable, is defined as follows:

7 $$ f(x) = \frac{1}{nh}\sum\limits_{i = 1}^{n} {K\left( {\frac{{\overline{x} - x_{i} }}{h}} \right)} $$

where $K$ is the kernel function, $X_{1} , \cdots X_{n}$ is the CCD of the cities in the sample, $\overline{x}$ is the mean value, $n$ is the number of sample observations, and $h$ is the window width. The article selects the Gaussian kernel function with high-precision reading to estimate the dynamic distribution level of the CCD of DF and RSD of the six major city clusters, and its functional expression is:

8 $$ K(x) = \frac{1}{{\sqrt {2\pi } }}e^{{\left( { - \frac{{x^{2} }}{2}} \right)}} $$

### Markov chains

Markov chains are employed to analyze the distribution and evolutionary trends of the CCD across different time frames and states, categorizing continuous values into discrete classes. Initially, a conventional Markov chain analysis dissects the internal trend characteristics of the system’s CCD. Here, the quartile method segments the CCD into four distinct levels: low, medium–low, medium–high, and high, based on their average values, facilitating the computation of the transition probability matrix. Subsequently, to incorporate spatial dynamics, a spatial Markov transition probability matrix (MTPX) is formulated following a spatial correlation assessment. Unlike the traditional Markov chain, the spatial Markov chain addresses the oversight of inter-regional interactions by integrating the concept of “spatial lag”. This approach involves calculating the weighted average attribute value of adjacent regions using a spatial weight matrix, thereby assessing the spatial lag condition of regional entities. This method, as Chen and Zhu^38^ note, enhances the analytical framework by considering the spatial dependencies and interactions among regions, offering a more comprehensive understanding of the CCD’s spatial and temporal evolution.

### Convergence analysis

#### $\sigma$-convergence

To assess the convergence trends of the CCD between DF and RSD across six major city clusters in China, this study employs the coefficient of variation to perform σ-convergence analysis. This involves examining the temporal trend of the CCD’s coefficient of variation both for the entire sample and within each of the six city clusters, to determine if regional disparities are diminishing over time. The coefficient of variation is calculated using the following formula:

9 $$ CV_{it} = \frac{{\sigma_{it} }}{{\overline{D}_{it} }} = \frac{{\sqrt {\frac{1}{{m_{i} }}\sum\limits_{m = 1}^{{m_{i} }} {(D_{imt} - \overline{D}_{it} )^{2} } } }}{{\overline{D}_{it} }} $$

Here, $\sigma_{it}$ represents the standard deviation of the CCD across the city clusters in year $t$, $D_{imt}$ denotes the coupling coordination level of the city $m$, region $i$ in year $t$, $m_{i}$ denotes the number of cities included in region $i$, and $\overline{D}_{it}$ denotes the average value of the CCD of all cities in region $i$ and year $t$.

#### $\beta$-convergence

In this study, $\beta$-convergence is utilized to explore the evolution of the CCD between DF and RSD across six major city clusters. $\beta$-convergence, stemming from neoclassical growth theory, is categorized into absolute and conditional $\beta$-convergence. The spatial correlation analysis conducted in this research indicates a substantial positive link between the DF and RSD coordination levels across these clusters. Conventional convergence theories often overlook the spatial interdependencies among regions. To address this gap, the current analysis incorporates an economic distance spatial weight matrix, enhancing the assessment of spatial convergence. Through rigorous testing, including Lagrange Multiplier (LM), Hausman, and Likelihood Ratio (LR) tests, the study opts for a two-way fixed-effects spatial Durbin model (SDM). This model is adept at evaluating both absolute and conditional $\beta$-convergence, providing a more nuanced understanding of how the CCD between DF and RSD evolves over time and space within the context of these city clusters. The SDM framework is specifically designed to capture the intricate spatial interactions, thereby offering a comprehensive analysis of convergence dynamics. The model is formulated as follows:

10 $$ \ln \left( {\frac{{q_{i,t + 1} }}{{q_{i,t} }}} \right) = \alpha + \beta \ln (q_{i,t} ) + \rho W_{ij} \ln \left( {\frac{{q_{i,t + 1} }}{{q_{i,t} }}} \right) + \gamma W_{ij} \ln (q_{i,t} ) + \mu_{i} + \eta_{t} + \varepsilon_{it} $$

11 $$ \begin{aligned} \ln \left( {\frac{{q_{i,t + 1} }}{{q_{i,t} }}} \right) = & \alpha + \beta \ln (q_{i,t} ) + \sum\limits_{z = 1}^{n} {\lambda_{z} X_{it} + } \rho W_{ij} \ln \left( {\frac{{q_{i,t + 1} }}{{q_{i,t} }}} \right) + \gamma_{1} W_{ij} \ln \left( {q_{i,t} } \right) \\ + \sum\limits_{z = 1}^{n} {\gamma_{2z} W_{ij} X_{it} } + \mu_{i} + \eta_{t} + \varepsilon_{it} \\ \end{aligned} $$

where $q_{it}$ denotes the CCD between DF and RSD of city $i$ in year $t$, and $\ln (\frac{{q_{i,t + 1} }}{{q_{i,t} }})$ denotes the growth rate , $\beta$ is the convergence coefficient, if $\beta < 0$ then it means that there is a convergence trend in the DF and RSD coupling coordination level across cities over time. Conversely, a positive $\beta$ signifies divergence. The rate of convergence is $\upsilon = - \ln (1 + \beta )/T$, $T$ represents the duration of the study, with the half-way convergence period $\tau = \ln (2)/\upsilon$. $\rho$ is the spatial lag coefficient, and $W_{ij}$ is the spatial weight matrix, here the economic distance matrix is selected as the spatial weight matrix, specifically using the GDP gap of each city as a measure of economic distance between cities. $\mu_{i} ,\;\eta_{t} ,\;\varepsilon_{it}$ represent city-fixed effects, time-fixed effects, and random error terms, respectively. $X_{it}$ is the selected control variables, referring to the existing literature^39^, the analysis framework of the factors influencing the CCD of DF and RSD is mainly constructed from five aspects: the level of economic growth, government intervention, financing constraints, software technology practitioners, and the level of informatization.

## Quadratic assignment procedure (QAP)

The concept of regional differences in CCD can be interpreted as a relational dynamic among regions, where variables are represented as relational data. These data are prone to autocorrelation and significant multicollinearity, making conventional statistical tests unsuitable for analysis^40^. This study adopts a resampling-based QAP to investigate the factors affecting regional CCD differences, aiming to uncover the underlying drivers. In the subsequent sections, we will delve into the relational data analysis framework, focusing on the establishment of a measurement model and the application of QAP.

### Model setting

The measurement model for relational data established in this study is articulated as follows:

12 $$ Y = \beta_{0} + \beta_{1} X + \varepsilon $$

In this model, $Y$ represents the dependent variable, signifying the regional differences in CCD. The term $\beta_{0}$, $\beta_{1}$ denotes the parameter set to be estimated, while $X$ corresponds to the explanatory variable, encapsulating the primary factors influencing regional CCD disparities. Lastly, $\varepsilon$ is the residual term, accounting for the variance in $Y$ not explained by $X$. The structural essence of this measurement model mirrors that of traditional attribute data models, with both employing a similar analytical framework. However, a distinct characteristic of the relational data model is its utilization of an n-order square matrix to represent the variables, highlighting the interconnected nature of the regional units under study. This format underscores the relational aspect of the data, enabling a nuanced analysis of the interdependencies and influences among regions.

### QAP

QAP is primarily employed for analyzing correlations within network structures^41^. Unlike traditional methods, QAP does not necessitate the assumption of variable independence, thereby circumventing issues of autocorrelation and multicollinearity inherent in data measurement models. A distinctive feature of relational data measurement models, as compared to conventional models, is the arrangement of variables in ordinal matrix form. This structural difference has led scholars to leverage the foundational principles and strengths of the QAP relational data analysis framework for constructing difference matrices to elucidate the genesis of disparities. QAP encompasses both correlation and regression analyses^42^. Correlation analysis within QAP is concerned with identifying the relationships between two matrices. In contrast, regression analysis within this framework seeks to understand the regression dynamics between multiple matrices and a single matrix, providing a comprehensive view of the interrelations and dependencies among the variables represented in these matrices.

## Evaluating the variability and temporal progression of CCD across regions

### CCD dynamics and regional variations

#### Assessment and examination of DF and RSD levels

The entropy value method is applied to measure the level of DF and RSD in 90 cities of six major city clusters from 2011 to 2020, and the results are shown in Table 2.

**Table 2:** Measurement of the level of DF and RSD (selected years).

Systems	Region	2011	2013	2015	2017	2019	2020	Average
DF	Full sample	0.1968	0.4520	0.5774	0.7231	0.7898	0.8169	0.5752
	Jing-jin-Ji	0.1904	0.4387	0.5530	0.6988	0.7609	0.7919	0.5534
	Yangtze River Delta	0.2234	0.4984	0.6301	0.7779	0.8714	0.9001	0.6305
	Pearl River Delta	0.2397	0.4939	0.6097	0.7582	0.8447	0.8693	0.6154
	Middle of Yangtze	0.1760	0.4378	0.5617	0.7100	0.7717	0.7992	0.5603
	Cheng-Yu	0.1820	0.4227	0.5515	0.6935	0.7358	0.7607	0.5422
	Beibu Gulf	0.1950	0.4263	0.5614	0.6992	0.7526	0.7766	0.5525
RSD	Full sample	0.2763	0.2920	0.3069	0.3183	0.3359	0.3425	0.3094
	Jing-jin-Ji	0.2729	0.2873	0.3011	0.3090	0.3209	0.3276	0.3011
	Yangtze River Delta	0.3491	0.3653	0.3730	0.3784	0.3972	0.4012	0.3753
	Pearl River Delta	0.3962	0.4247	0.4354	0.4510	0.4550	0.4560	0.4345
	Middle of Yangtze	0.2425	0.2547	0.2731	0.2884	0.3052	0.3126	0.2766
	Cheng-Yu	0.2122	0.2279	0.2467	0.2577	0.2835	0.2935	0.2505
	Beibu Gulf	0.2220	0.2366	0.2550	0.2676	0.2930	0.3016	0.2595

Observing the trajectory of DF progress within each city cluster over the years, the level of DF advancement experienced a remarkable surge during the examined period, exhibiting an overarching enhancement of 315.09% over the span of a decade. On a sub-regional level, the Yangtze River Delta showcased the most substantial progress, amounting to an impressive 354.09%, while the Beibu Gulf displayed the most modest improvement, with a growth rate of 298.26%. The collective average of DF among all sampled cities stands at 0.5752, with an average annual growth rate of 17.13% throughout the assessment period. Notably, DF experienced a sharp increase from 2011 to 2013. Possible reasons for this include the introduction and rapid expansion of digital financial services in China during the early 2010s. Companies such as Alipay and WeChat Pay began to gain significant traction during this period, leading to a substantial increase in digital financial transactions and services. Simultaneously, the Chinese government introduced several policies aimed at promoting inclusive finance and the development of DF. These policies included regulatory support for fintech companies and initiatives to enhance financial accessibility in rural and underserved areas, contributing to the rapid growth of DF.

Regarding the realm of RSD, the pace of growth remained sluggish throughout the examined period, with both the overall sample and individual city clusters displaying low growth rates. Over the course of a decade, the overall sample as a whole witnessed a modest expansion of 23.96%. In terms of sub-regions, Cheng-Yu exhibited the highest level of improvement, with a commendable growth rate of 38.31%, while Yangtze River Delta showcased the lowest progress at a mere 14.92%. Throughout the ten years, the comprehensive average value of the full sample amounted to 0.3094, with Pearl River Delta boasting the highest mean value of 0.4345, and Cheng-Yu registering the lowest mean value of 0.2505. The study’s findings emphasize the evident inter-regional disparities in the level of RSD among the six major city clusters, with the overall conclusion suggesting that the level of DF development in each city cluster surpasses that of RSD.

#### System CCD outcomes and evaluation

Utilizing the CCD model as a foundation to assess the CCD of DF and RSD within the six city clusters, an examination of the interplay and coordination between DF and RSD is conducted for both the collective city clusters and individual clusters throughout 2011 to 2020. The comprehensive findings, illustrating the outcomes, are presented in Table 3.

**Table 3:** The CCD in the six major city clusters (selected years).

Region	Time	2011	2013	2015	2017	2019	2020	Average
Full sample	CCD	0.4783	0.5974	0.6441	0.6884	0.7138	0.7233	0.6365
	Grade	Near non	Near	Primary	Primary	Intermediate	Intermediate	Primary
Jing-jin-Ji	CCD	0.4742	0.5918	0.6354	0.6789	0.7002	0.7110	0.6272
	Grade	Near non	Near	Primary	Primary	Intermediate	Intermediate	Primary
Yangtze River Delta	CCD	0.5258	0.6502	0.6936	0.7343	0.7645	0.7726	0.6859
	Grade	Near	Primary	Primary	Intermediate	Intermediate	Intermediate	Primary
Pearl River Delta	CCD	0.5516	0.6720	0.7136	0.7606	0.7837	0.7900	0.7069
	Grade	Near	Primary	Intermediate	Intermediate	Intermediate	Intermediate	Intermediate
Middle of Yangtze	CCD	0.4524	0.5757	0.6238	0.6707	0.6948	0.7050	0.6165
	Grade	Near non	Near	Primary	Primary	Primary	Intermediate	Primary
Cheng-Yu	CCD	0.4413	0.5552	0.6053	0.6485	0.6743	0.6849	0.5973
	Grade	Near non	Near	Primary	Primary	Primary	Primary	Near
Beibu Gulf	CCD	0.4534	0.5601	0.6120	0.6554	0.6827	0.6925	0.6047
	Grade	Near non	Near	Primary	Primary	Primary	Primary	Primary

The CCD of the full sample and each city cluster has exhibited a steady augmentation throughout the examined period. Notably, the CCD of the full sample has undergone a significant transformation, transitioning from a state of near non-coordination in 2011 to an intermediate coordination level by 2020. As of 2020, except Cheng-Yu and Beibu Gulf, all other city clusters have attained an intermediate level of coordination. When considering sub-regions, both Yangtze River Delta and Pearl River Delta consistently surpass the average CCD of the full sample across all years, while the CCD of Jing-jin-Ji remains closest to the aforementioned average, albeit slightly lower. Amongst the city clusters, Cheng-Yu exhibits the lowest level of coupling coordination, with an average value of 0.5973 throughout the observation period, whereas the remaining city clusters all boast an average value of coupling coordination exceeding 0.600.

### Dynamics of CCD distribution evolution

#### Kernel density estimation

**Figure 4:**
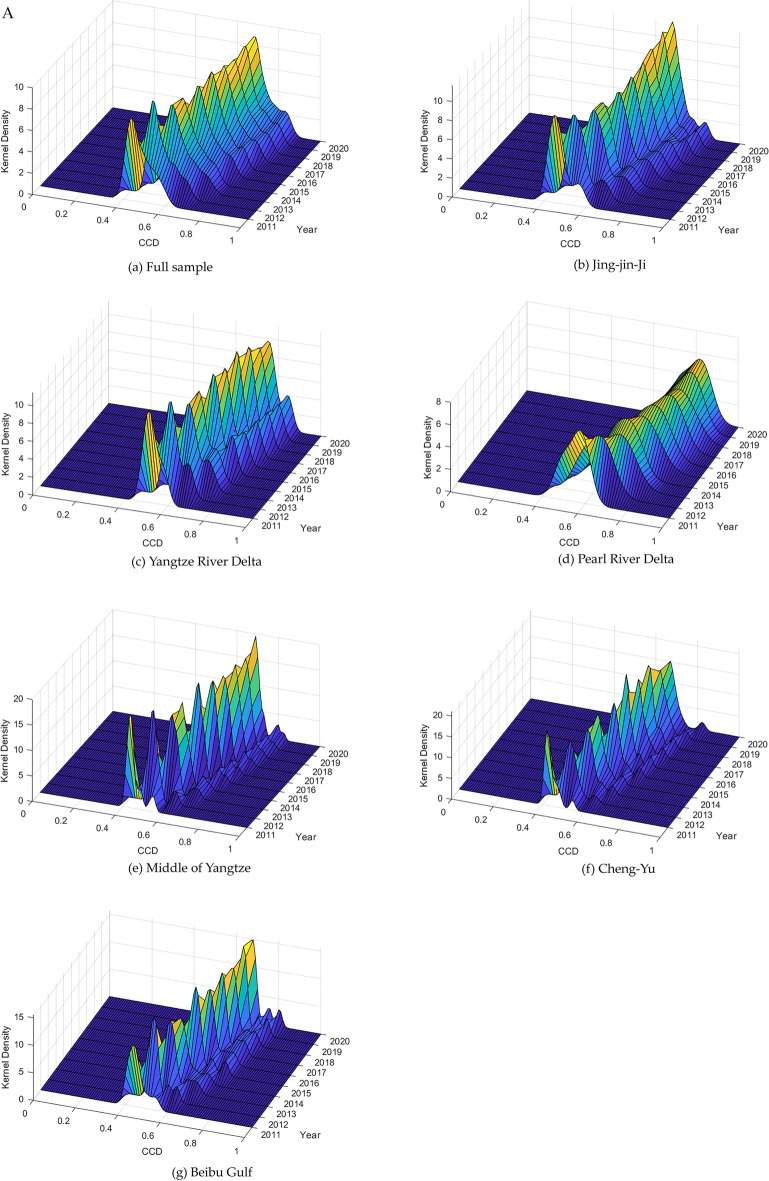
Kernel density estimation of the CCD.

Due to variations in factor endowments and stages of development among the six city clusters, the measured outcomes and trends for each cluster can differ over time. Therefore, this study utilizes kernel density estimation^43^ to analyze the dynamic evolution of CCD across the entire sample and within the individual city clusters. This analysis encompasses an exploration of distribution location and distribution pattern. The findings of this analysis are visually presented in Fig. 4.

Figure 4(a) illustrates the dynamic evolution of CCD across the entire sample. The distribution’s central point and range have progressively shifted rightward, though the rate of this shift decreased annually, eventually plateauing post-2017. Regarding the distribution pattern, the primary peak of the curve has consistently risen, depicting an M-shaped evolution with phases of increase, decline, subsequent rise, and then stabilization. Additionally, the narrowing of the primary peak suggests a growing centralization in the coupling coordination level across the sample, indicative of diminishing regional disparities.

Figure 4(b) through (g) display the dynamic evolutionary trends of CCD across different regions: Jing-Jin-Ji, Yangtze River Delta, Pearl River Delta, Middle of Yangtze, Cheng-Yu, and Beibu Gulf, during the study period. Initially, regarding the distribution location, all six city clusters, along with the overall sample, exhibit a primarily upward trend, albeit with a minor regression between 2018 and 2020. Secondly, concerning the distribution pattern, all city clusters, with the exception of the Pearl River Delta, developed dual peaks, signifying an increase in regional disparities in CCD changes.

#### Markov chain analysis

This study delves into the internal dynamics and spatial transfer characteristics of CCD by employing the Markov Transfer Probability Matrix (MTPX) for analysis. Initially, the conventional Markov chain method is applied to assess the inherent trend characteristics of CCD within the system. The cities sampled are categorized into four CCD levels—low, medium–low, medium–high, and high—using the quartile method, based on their average magnitudes. Table 4 presents the resulting transfer probability matrix, where the diagonal elements notably exceed the non-diagonal ones. Specifically, the probabilities of maintaining the same CCD level after one year are 63.11% for low, 61.43% for medium–low, 72.73% for medium–high, and 99.43% for high-level areas. This pattern suggests a stable degree of coupling coordination, leading to a ‘club convergence’ effect. Moreover, the convergence probability is marginally higher at the low and high levels than at the medium–low and medium–high levels, indicating a “Matthew effect” in the system’s coupling coordination.

**Table 4:** Traditional MTPX for the CCD.

Type	Low	Mid-to-low	Mid-to-high	High	No. of observations
Low	0.6311	0.3600	0.0089	0.0000	225
Mid-to-low	0.0000	0.6143	0.3857	0.0000	223
Mid-to-high	0.0000	0.0000	0.7273	0.2727	187
High	0.0000	0.0000	0.0057	0.9943	175

#### Spatial Markov chain analysis

The study computes the spatial Moran’s I index for CCD from 2011 to 2020 across various city clusters using Stata 17.0, based on the collected CCD data, as shown in Table 5. The analysis consistently shows a positive global Moran’s I index for the CCD in the six major city clusters during the study period. This positive index indicates that CCD levels in one region are correlated with those in neighboring areas, resulting in a spatial pattern of high-high and low-low clusters. The Moran’s I index values, ranging from 0.3 to 0.5, demonstrate a stable spatial positive correlation in CCD levels across the regions.

**Table 5:** Global Moran’s index for CCD (specific years).

	2011	2013	2015	2017	2019	2020
Moran’s I	0.477***	0.442***	0.423***	0.428***	0.421***	0.390***
Z-value	6.093	5.672	5.428	5.496	5.413	5.017

Due to spatial constraints, this document only reports measurements for select years. A notation of “***” indicates a statistical significance at the 1% level.

The above findings underscore the necessity of incorporating spatial factors in constructing the spatial MTPX, as illustrated in Table 6. Notably, variability is evident across the four transfer probability matrices for different spatial lag types, indicating that the likelihood of shifts in coupling coordination is affected by the neighboring regions’ coordination levels. Moreover, the diagonal elements in the matrices do not always dominate the non-diagonal ones, suggesting a reduced predictability of sustained coupling coordination due to spatial spillover effects. The presence of non-zero elements adjacent to the diagonal signifies the potential for both upward and downward transitions in CCD levels, reflecting inherent instability. Transitions tend to occur between neighboring levels, with cross-level transfers being less common. Additionally, the influence of spatial lag varies with the level; for example, under medium–high level lag, the transition probabilities from low to medium–low are notably higher than those under low-level lag. Specifically, transition probabilities decrease from 75% at the low level to 54.41% and 15.73% at medium–low and medium–high levels respectively, under medium–high lag conditions. This pattern underscores that transition probabilities are determined by both the lag type and the initial level of coupling coordination.

**Table 6:** Spatial MTPX for the CCD.

Type of lag	t/(t + 1)	I	II	III	IV	No. of observations
Low	I	0.7045	0.2841	0.0114	0.0000	176
	II	0.0000	0.4333	0.5667	0.0000	30
	III	0.0000	0.0000	0.4000	0.6000	10
	IV	0.0000	0.0000	0.0000	1.0000	1
Mid-to-low	I	0.3778	0.6222	0.0000	0.0000	45
	II	0.0000	0.7667	0.2333	0.0000	120
	III	0.0000	0.0000	0.5938	0.4063	32
	IV	0.0000	0.0000	0.0000	1.0000	26
Mid-to-high	I	0.2500	0.7500	0.0000	0.0000	4
	II	0.0000	0.4559	0.5441	0.0000	68
	III	0.0000	0.0000	0.8427	0.1573	89
	IV	0.0000	0.0000	0.0222	0.9778	45
High	I	0.0000	0.0000	0.0000	0.0000	0
	II	0.0000	0.2000	0.8000	0.0000	5
	III	0.0000	0.0000	0.6786	0.3214	56
	IV	0.0000	0.0000	0.0000	1.0000	103

## Convergence analysis

### $\sigma$-convergence result analysis

**Figure 5:**
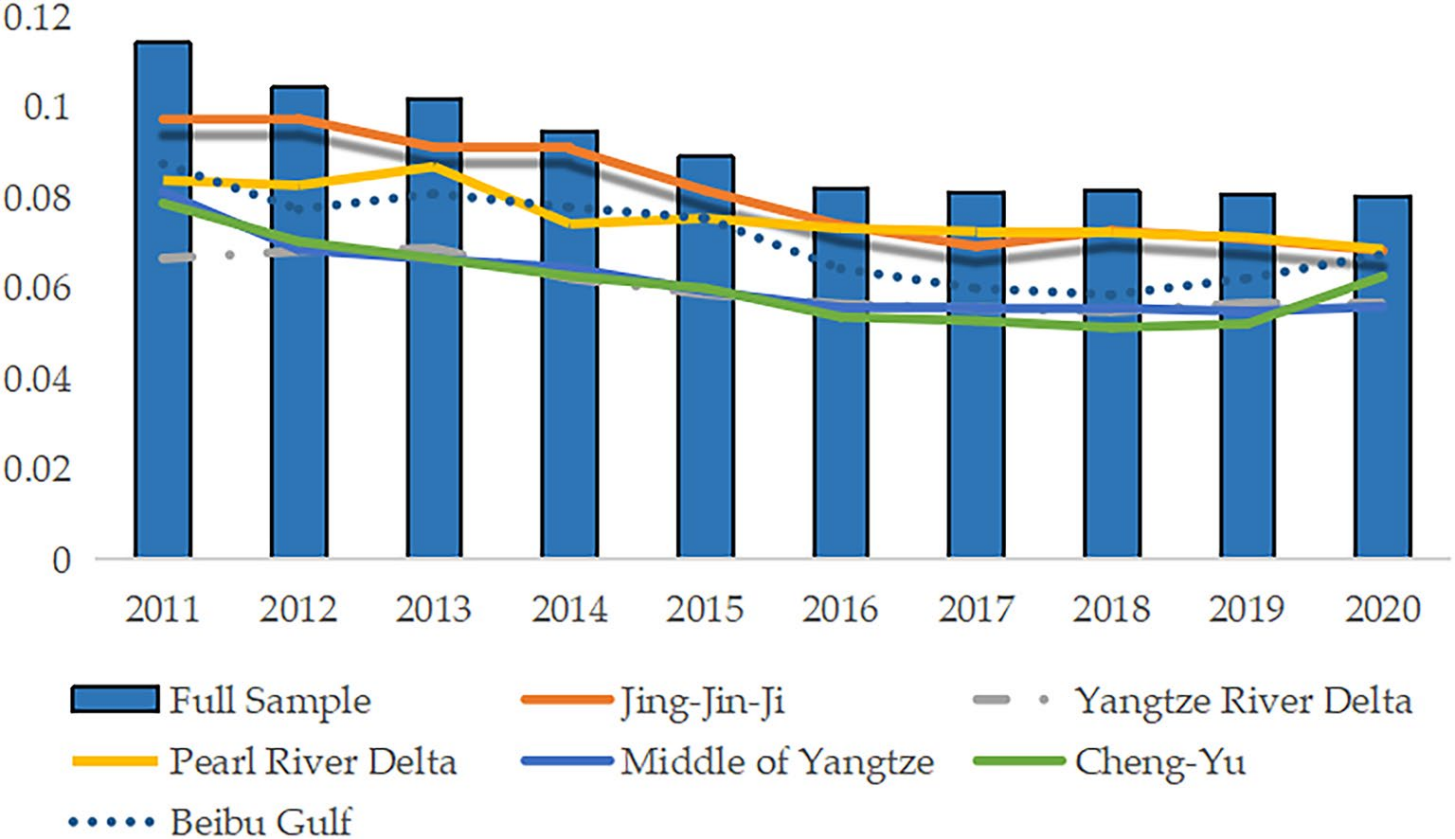
Visual depiction of CCD $\sigma$-convergence.

This study uses the coefficient of variation to perform a $\sigma$-convergence analysis, assessing the variation in CCD levels from 2011 to 2020.The resulting convergence trend, depicted in Fig. 5, reveals a consistent and gradual decline in the CCD coefficient, indicating a notable convergence at the national level. Notably, the Jing-Jin-Ji region exhibits the most substantial decrease in the coefficient, closely aligning with the convergence trend observed in the full sample. Pearl River Delta, Yangtze River Delta, and Beibu Gulf regions demonstrate the second-largest reduction in coefficients, albeit with slightly weaker convergence. Conversely, the Middle of Yangtze and Cheng-Yu regions display the smallest decrease in coefficients, coupled with a relatively low rate of convergence.

### $\beta$-convergence result analysis

#### Absolute $\beta$-convergence analysis

The significant positive spatial correlation among CCDs, as previously discussed, necessitates considering spatial factors when analyzing CCD convergence. The LM test indicates that the spatial Durbin model (SDM) is more valid. Furthermore, the Hausman test rejects the notion of random effects, leading to the selection of a two-way fixed-effect SDM to evaluate the $\beta$-convergence of CCD.

Table 7 shows the absolute $\beta$-convergence test results for the overall sample and the six city clusters. The negative sign of the estimated coefficients across all regions indicates absolute $\beta$-convergence in CCD. The rates and time frames of convergence differ among regions. Specifically, the convergence rates for the entire sample and the six key city clusters are 8.7%, 4.68%, 6.48%, 5.39%, 14.17%, 11.92%, and 7.44%, respectively. Correspondingly, the half-way convergence period for these regions are 7.9 years, 14.81 years, 10.70 years, 12.86 years, 4.89 years, 5.82 years, and 9.31 years. The Middle of Yangtze and Cheng-Yu clusters show faster convergence than the overall average, with the Middle of Yangtze achieving the highest speed and the shortest half-way period of 4.89 years. Additionally, the conditional $\beta$-convergence for both the full sample and the individual clusters was analyzed, taking into account various control variables to reflect the actual developmental dynamics.

**Table 7:** Absolute $\beta$-convergence of the CCD.

Variables	Full sample	Jing-jin-Ji	Yangtze River Delta	Pearl River Delta	Middle of Yangtze	Cheng-Yu	Beibu Gulf
$\beta$	− 0.5449*** (− 23.34)	− 0.3437*** (− 5.66)	− 0.4419*** (− 8.16)	− 0.3845*** (− 4.59)	− 0.7207*** (− 18.01)	− 0.6578*** (− 9.16)	− 0.4883*** (− 6.38)
Time fixed	Yes	Yes	Yes	Yes	Yes	Yes	Yes
City fixed	Yes	Yes	Yes	Yes	Yes	Yes	Yes
R2	0.544	0.1298	0.6751	0.6101	0.7383	0.7366	0.7143
Convergence Rate	8.7	4.68	6.48	5.39	14.17	11.92	7.44
Half-way Convergence Period	7.9	14.81	10.70	12.86	4.89	5.82	9.31
N	810	108	162	81	243	126	90

Note: The symbols *, **, and *** denote significance levels of 10%, 5%, and 1%, respectively. T-statistics are provided in parentheses.

#### Conditional $\beta$-convergence analysis

To analyze the factors that may affect the CCD, this paper introduces the following control variables: (1) Economic growth. This paper measures the level of economic development of each region by GDP per capita, and the data is taken as a logarithm. (2) Government intervention. Government fiscal expenditure as a share of GDP. (3) Financing constraints. Measured by the ratio of deposit and loan balances of financial institutions to GDP. (4) Software practitioners. Measured by the ratio of the number of employees in information transmission, computer services, and software to the average annual population. (5) Informatization level. The number of international Internet users is taken as a logarithm.

Table 8 presents the results of spatial conditional $\beta$-convergence, factoring in the control variables mentioned earlier. The coefficients for both the entire sample and the six major city clusters show significant negative values, indicating a pronounced $\beta$-convergence in CCD. The recorded convergence rates are 10.02%, 6.82%, 8.55%, 12.70%, 16.01%, 17.74%, and 9.21%, respectively. The corresponding half-way convergence times are 6.91, 10.17, 10.11, 5.46, 4.33, 3.91, and 7.53 years. Notably, after including the control variables, there is an observable increase in convergence speed for all areas, alongside a reduction in the half-life periods. This suggests that the control variables significantly enhance the $\beta$-convergence rate in CCD across the sample and the six city clusters. Moreover, the order of convergence speeds alters, with Cheng-Yu, Middle of Yangtze, and Pearl River Delta leading, followed by the full sample, Beibu Gulf, Yangtze River Delta, and Jing-jin-Ji.

**Table 8:** Conditional $\beta$-convergence of the CCD.

Variables	Full sample	Jing-jin-Ji	Yangtze River Delta	Pearl River Delta	Middle of Yangtze	Cheng-Yu	Beibu Gulf
β	− 0.5943*** (− 25.00)	− 0.4585*** (− 5.80)	− 0.5366*** (− 8.81)	− 0.6812*** (− 9.07)	− 0.7633*** (− 18.56)	− 0.7974*** (− 10.14)	− 0.5635*** (− 6.70)
Economic growth	0.0062* (1.79)	0.0027 (0.25)	0.0117* (1.66)	0.0183*** (4.05)	0.0184** (2.00)	− 0.0185* (− 1.76)	0.0200 (1.36)
Government intervention	0.0278** (2.22)	0.0463 (0.78)	− 0.0437 (− 0.67)	0.0565 (0.88)	0.1107** (2.11)	− 0.0106 (− 0.63)	0.1630** (2.35)
Financing constraints	− 0.0025** (− 2.37)	0.0025 (0.54)	− 0.0025 (− 0.83)	− 0.0125*** (− 3.40)	− 0.0062 (− 1.02)	− 0.0008 (− 0.13)	− 0.0030 (− 1.59)
Software practitioners	− 0.4987*** (− 3.64)	− 0.4777 (− 1.03)	− 0.2634 (− 1.40)	− 0.3198* (− 1.70)	− 1.2889* (− 1.84)	− 1.487*** (− 3.81)	− 0.7284 (− 0.23)
Informatization level	0.0035** (2.53)	0.0060 (1.42)	0.0036 (1.44)	− 0.0016 (− 0.41)	0.0005 (0.11)	0.0072* (1.88)	0.0032 (0.80)
Time fixed	Yes	Yes	Yes	Yes	Yes	Yes	Yes
City fixed	Yes	Yes	Yes	Yes	Yes	Yes	Yes
R2	0.6108	0.5032	0.7174	0.5927	0.7393	0.7064	0.6555
Convergence Rate	10.02	6.82	8.55	12.70	16.01	17.74	9.21
Half-way Convergence Period	6.91	10.17	8.11	5.46	4.33	3.91	7.53
N	810	108	162	81	243	126	90

Note: The symbols *, **, and *** denote significance levels of 10%, 5%, and 1%, respectively. T-statistics are provided in parentheses.

## QAP analysis

### QAP correlation analysis

In this paper, we employ the Ucinet software to conduct 2000 random permutations, yielding the outcomes of QAP correlation analysis concerning the disparities in CCD among regions and the explanatory variables. These results are meticulously presented in Table 9. It is revealed that the correlation coefficients between the regional dissimilarities in CCD and the regional disparities in economic growth, the magnitude of financial industry, the proportion of telecommunication business, the degree of urbanization, market openness, and advancement of industrial structure are all positively inclined. Furthermore, the correlation coefficients of the remaining variables attain statistical significance at the 1% level, excluding the proportion of telecommunication enterprises. This observation implies that alterations in the development of any of these factors give rise to modifications in CCD disparities. Specifically, the correlation coefficients between the regional dissimilarities in economic growth, the magnitude of the financial industry, the degree of urbanization, market openness, and advancement of industrial structure, and the regional disparities in CCD are recorded as 0.889, 0.769, 0.931, 0.856, and 0.505 respectively, in the aforementioned sequence.

**Table 9:** QAP correlation analysis results.

Variables	CCD	Economic growth	Magnitude of financial industry	Proportion of telecommunication business	Urbanization	Market openness	Advancement of industrial structure
CCD	1.000***	–	–	–	–	–	–
Economic growth	0.889***	1.000***	–	–	–	–	–
Magnitude of financial industry	0.769***	0.647***	1.000***	–	–	–	–
Proportion of telecommunication business	0.095	− 0.102	0.211**	1.000***	–	–	–
Urbanization	0.931***	0.880***	0.762***	0.073	1.000***	–	–
Market openness	0.856***	0.780***	0.609***	-0.062	0.763***	1.000***	–
Advancement of industrial structure	0.505***	0.285***	0.675***	0.277**	0.421***	0.345***	1.000***

The symbols *, **, and *** denote significance levels of 10%, 5%, and 1%, respectively.

### QAP regression analysis

To assess the impact and direction of six identified factors, we conducted a QAP regression with 2000 permutations for random replacement. This analysis used the matrix of regional differences in coupling coordination as the dependent variable and the matrices of the six factors as explanatory variables, with results displayed in Table 10. The adjusted R-squared value of 0.946 suggests that these matrices account for 94.6% of the variation in regional CCD differences. The regression analysis reveals that standardized coefficients, which negate scale effects, allow for direct comparison across variables. Specifically, the financial industry’s impact on CCD differences is negligible (-0.008, not significant), indicating it is not a current driver of regional CCD discrepancies. In contrast, significant positive standardized coefficients for economic growth, telecommunication business proportion, urbanization, market openness, and industrial structure advancement indicate their substantial roles in creating regional CCD variations. Urbanization has the most significant impact, with a coefficient of 0.409, demonstrating that increased urbanization disparity leads to greater CCD regional differences. The influence hierarchy of other factors, in descending order, includes market openness (0.289), economic growth (0.276), industrial structure advancement (0.139), and telecommunication business proportion (0.074).

**Table 10:** Results of QAP regression analysis on factors influencing regional CCD disparities.

Variables	Unstandardized regression coefficient	Standardized regression coefficient	Probability of significance	Probability A	Probability B
Intercept	0.000	0.000			
Economic growth	0.030	0.276	0.000	0.000	1.000
Magnitude of financial industry	− 0.069	− 0.008	0.447	0.554	0.447
Proportion of telecommunication business	0.526	0.074	0.007	0.007	0.994
Urbanization	0.002	0.409	0.000	0.000	1.000
Market openness	0.010	0.289	0.000	0.000	1.000
Advancement of industrial structure	0.015	0.139	0.000	0.000	1.000

## Discussion

This study establishes an evaluation framework for DF and RSD within the context of city clusters. It assesses the development of 90 cities across six major Chinese city clusters from 2011 to 2020 using the entropy value method and evaluates the CCD levels of DF and RSD in these clusters through the CCD model. The research further explores the regional disparities, convergence, and dynamic changes in CCD by employing methods such as kernel density estimation, Markov chain analysis, and spatial convergence models. The paper concludes with an empirical analysis of the factors affecting regional CCD variations, using the QAP method. This comprehensive analysis sheds light on the coupling and coordination dynamics between DF and RSD in China’s city clusters, providing insights into the ongoing progress of RSD and urbanization. Additionally, the findings offer a theoretical basis and policy guidance to enhance the CCD integration of DF and RSD in the current and forthcoming phases.

Our analysis reveals that both DF and RSD experienced growth during the observation period from 2011 to 2020. However, the pace of development in DF significantly outstripped that of RSD. This discrepancy may be attributed to several key factors. Firstly, the rapid advancement in information technologies such as big data, artificial intelligence, and 5G has catalyzed the explosive growth of DF. These technologies have enabled more efficient, scalable, and innovative financial services that cater to a broad range of needs, thus driving the swift expansion of DF across various regions. In contrast, RSD is a multifaceted concept encompassing economic, health, and green development, among other aspects. The development of RSD requires coordinated efforts across multiple domains, including sustainable economic practices, environmental protection, and social well-being. This comprehensive approach inherently involves complex interactions and dependencies among diverse factors, which can slow down overall progress. Moreover, the varying resource endowments and talent conditions across different regions further contribute to the uneven development of RSD. For instance, regions with abundant natural resources or advanced technological infrastructure might progress faster in certain aspects of RSD, while others lag due to less favorable conditions. These inter-regional disparities highlight the challenges in achieving uniform sustainable development, contrasting with the relatively more homogeneous growth observed in DF.

During the decade-long period, the Yangtze River Delta demonstrated the most substantial progress in DF, achieving an impressive 354.09% increase, while the Beibu Gulf exhibited the least improvement with a 298.26% growth rate. On average, DF across all sampled cities grew by 315.09%, with an annual growth rate of 17.13%, underscoring the rapid evolution of the DF sector. Conversely, RSD growth remained sluggish throughout the same period. The overall sample saw a modest 23.96% increase, with sub-regional disparities evident. The Cheng-Yu region achieved the highest growth rate in RSD at 38.31%, while the Yangtze River Delta recorded the lowest progress at 14.92%. The Pearl River Delta had the highest mean value of RSD at 0.4345, whereas Cheng-Yu had the lowest at 0.2505, indicating significant regional differences. These findings align with the study by An et al.^44^, which indicates that while DF has benefited from rapid technological advancements, the comprehensive nature of RSD requires more time and coordinated efforts to achieve substantial progress. The development of DF surpasses that of RSD, reflecting the easier scalability and faster adoption of DF technologies compared to the multifaceted and region-specific challenges associated with sustainable development.

The results of this study indicate that the CCD of the full sample and each city cluster has exhibited a steady increase throughout the examined period. As shown in Table 3, the CCD values during the observation period are all greater than 0.4. Our findings on CCD align with those of Li et al.^14^, who studied the CCD level between the green finance system and sustainable development across 30 provinces in China. Their results revealed that the CCD between the green finance system and sustainable development is greater than 0.4, indicating no imbalance and suggesting that green finance and sustainable development in China are complementary.

From the standpoint of regional differences, the six major city clusters exhibit profound differences in the CCD of DF and RSD, showcasing notable hierarchical characteristics. These differences can be classified into three distinct tiers. The Pearl River Delta stands at the apex as the first tier, boasting the highest level of coupling and coordination, consistently surpassing the average level of the full sample throughout the specified period. The second tier comprises Jing-Jin-Ji and the Yangtze River Delta, whose development levels align with the average level of the full sample. The third tier includes the Middle of Yangtze, Cheng-Yu, and Beibu Gulf, all of which exhibit an overall lower level of coupling coordination, consistently falling below the average level of the full sample during the specified period.

The Pearl River Delta has historically been one of China’s most economically dynamic and developed areas. It benefits from a robust industrial base, significant foreign investment, and advanced infrastructure, which support both DF and RSD. The high level of economic activity in this region naturally facilitates better coupling and coordination between DF and RSD. In contrast, the Jing-Jin-Ji and Yangtze River Delta regions, while also economically advanced with substantial industrial and technological bases, exhibit growth rates and levels of DF and RSD integration that align with the average level of the full sample but do not match the rapid pace of the Pearl River Delta. This discrepancy may be due to different industrial structures or slightly slower economic growth rates. The third-tier regions, including the Middle of Yangtze, Cheng-Yu, and Beibu Gulf, generally have less developed economies compared to the first and second tiers. These regions face greater challenges in terms of infrastructure, investment, and economic diversification, which can impede the effective coupling and coordination of DF and RSD. Addressing these disparities requires tailored regional strategies that consider the unique strengths and challenges of each cluster to foster balanced growth in DF and RSD.

The research perspective adopted in this paper is innovative, focusing on the examination of the coupled and coordinated relationship between DF and RSD from the standpoint of city clusters. This particular area of study remains relatively unexplored, making it noteworthy. For instance, Yang and Tang^45^ researched this subject using a sample of 13 cities in Jing-Jin-Ji, and their findings demonstrated a consistent year-on-year increase in the CCD between DF and RSD, aligning with the conclusions drawn in this paper.

In addition to addressing the above questions, this study provides several new contributions to the relevant research. Firstly, the research perspective offers a new quantitative analysis viewpoint. This study is the first to use the CCD model to analyze the relationship between DF and RSD in China’s six major city clusters. This analysis helps the government and academia better understand the coordinated development relationship between DF and RSD. Secondly, it further analyzes the evolution trends and influencing factors of the CCD spatial pattern of DF and RSD, providing a basis for the government to formulate policies that promote the coordinated development of DF and RSD.

Regarding the specific applications of the research conclusions and policy implications, the study highlights several critical insights. The identification of primary drivers behind regional CCD disparities—such as economic growth, the proportion of telecommunication business, urbanization level, market openness, and the degree of industrial structure advancement—provides a basis for targeted policy interventions. Policymakers can use these insights to design strategies that promote balanced and coordinated development between DF and RSD across different regions. By categorizing the six major city clusters into three distinct tiers based on their CCD levels, our study offers a practical framework for regional development planning. This hierarchical classification can guide resource allocation and development priorities, helping regions at different development stages to enhance their coupling coordination. Additionally, the analysis of $\sigma$-convergence and $\beta$-convergence underscores the importance of economic growth and other control variables in accelerating the convergence process. Policymakers can leverage these findings to implement measures that facilitate faster convergence and reduce regional disparities, fostering more equitable and sustainable development. These contributions and insights not only advance the academic understanding of the relationship between DF and RSD but also provide practical guidance for policymakers aiming to promote regional development and sustainability.

Despite these contributions, this study also has certain limitations. Firstly, although the latest available data was used, there is still some lag in data updates. Future research should use more updated data to improve the accuracy of the results. Secondly, while this paper focuses solely on the six national city clusters in China, it should be acknowledged that China’s vast geographical expanse encompasses numerous smaller city clusters, each playing a pivotal role in regional economic development. For instance, Mao et al.^46^conducted a study encompassing 19 city clusters in China, shedding light on the significance of expanding the research sample scope. By enlarging the scope of the research sample, we can gain a more profound and comprehensive understanding of the coupling and coordination of DF and RSD across diverse regions of China. Future research should expand the sample and broaden the scope of data selection.

## Conclusions and policy implications

### Conclusions

This study develops an evaluation index for DF and RSD, assessing the progress of China’s six principal city clusters between 2011 and 2020. It delves into the regional disparities, dynamic changes, and convergence patterns of the CCD levels. Key findings are summarized below.

Firstly, the CCD between DF and RSD has exhibited a consistent and progressive increase in both the full sample and the six major city clusters. When considering regional differences, the six major city clusters demonstrate notable differences in the CCD between DF and RSD, showcasing a significant hierarchical pattern, resulting in a division into three distinct tiers.

Secondly, kernel density estimation reveals dynamic distribution changes in the CCD between DF and RSD across the six major city clusters. The analysis indicates a consistent rightward shift in the central position and range of the distribution curve during the study period, with the shift’s momentum decreasing annually and halting after 2017. Regarding the distribution’s shape, the primary peak’s height progressively rises, forming an M-shaped evolutionary trajectory marked by periods of increase, decrease, resurgence, and eventual stabilization.

Thirdly, examining convergence characteristics reveals both β-convergence and spatial convergence in the CCD dynamics between DF and RSD across the full sample and the six major city clusters. The β-convergence analysis indicates a consistent annual decline in the coefficient, signifying a steady convergence across all regions. Spatial convergence analysis reveals both absolute and conditional convergence within these areas. With the inclusion of economic growth level and other control variables, the convergence rates for both the full sample and the six city clusters have variedly increased, shortening the time to reach halfway convergence. The sequence of convergence speed, from fastest to slowest, is Cheng-Yu, Middle of Yangtze, Pearl River Delta, full sample, Beibu Gulf, Yangtze River Delta, and Jing-Jin-Ji.

Fourthly, the primary drivers of regional CCD disparities are variations in economic growth, the proportion of telecommunication business, urbanization level, market openness, and the degree of industrial structure advancement. Conversely, disparities in the financial industry’s size have a negligible impact on CCD’s regional differences.

### Policy implications

Based on the above conclusions, the following policy recommendations are proposed to promote the coordinated development of different city clusters.

Firstly, accelerate the coordinated development of each city cluster. There is an imbalance between the six major city clusters in terms of the CCD, with Pearl River Delta having a relatively high level of DF and RSD, and the CCD consistently higher than the full sample. Middle of Yangtze, Cheng-Yu, and Beibu Gulf have lagged to different degrees in terms of the level of DF and RSD, and the CCD is still somewhat different from the average of the full sample. Therefore, the location advantages and resource conditions of different city clusters should be fully utilized to promote the coordinated development of their level of DF and RSD, to promote the steady improvement of the CCD.

Secondly, it is imperative to devise region-specific developmental policies. The six major city clusters exhibit distinct geographic locations, resource endowments, economic prowess, and business environments, thereby presenting varied advantages and shortcomings in terms of DF and economic as well as social progress. Hence, it becomes indispensable to establish tailored developmental policies that align with the developmental level and structural characteristics of each city cluster. These policies aim to facilitate the cultivation of sustainable economic and social growth, while fostering a continuous enhancement of the DF level. Moreover, they seek to propel the realization of the regional rebalancing and development strategy, predicated on the continual augmentation of the coupling and coordination between DF and RSD.

Therefore, it is imperative to devise region-specific development policies tailored to the developmental stage and structural attributes of each city cluster. These policies should aim to foster sustainable economic and social growth, enhance the level of DF, and facilitate regional rebalancing and development strategies. This approach should be underpinned by a continual enhancement of the coupling and coordination between DF and RSD. Moreover, advancing these strategies will be crucial for improving the synergy between DF and high-quality economic and social development, aligning with goals for balanced regional progress.

Thirdly, the primary factors contributing to regional differences in the CCD stem from variations in economic growth, the proportion of telecommunication business, urbanization level, the level of market openness, and differences in advancement of industrial structure. It is imperative for each city cluster to continuously narrow the regional differences resulting from these influencing factors during their developmental journey. Tailored policy measures should be formulated to address the shortcomings specific to their own development, expediting the remediation of weaknesses and consistently diminishing the regional disparities with other city clusters. In doing so, a rapid elevation of the level of coupling and coordination between DF and RSD can be achieved.

## Data Availability

The datasets used during the current study available from the corresponding author on reasonable request.
